# Identities associated with Milne–Thomson type polynomials and special numbers

**DOI:** 10.1186/s13660-018-1679-x

**Published:** 2018-04-13

**Authors:** Yilmaz Simsek, Nenad Cakic

**Affiliations:** 1 Department of Mathematics, Faculty of Science, University of Akdeniz, Antalya, Turkey; 2 Department of Mathematics, Faculty of Electrical Engineering, University of Belgrade, Belgrade, Serbia

**Keywords:** 05A15, 05A10, 11B68, 11B83, Generating function, Functional equation, Bernoulli numbers and polynomials, Euler numbers and polynomials, Stirling numbers, Array polynomials, Milne–Thomson polynomials, Hermite polynomials, Central factorial numbers, Cauchy numbers, Special functions, *p*-adic integral

## Abstract

The purpose of this paper is to give identities and relations including the Milne–Thomson polynomials, the Hermite polynomials, the Bernoulli numbers, the Euler numbers, the Stirling numbers, the central factorial numbers, and the Cauchy numbers. By using fermionic and bosonic *p*-adic integrals, we derive some new relations and formulas related to these numbers and polynomials, and also the combinatorial sums.

## Introduction

Recently, many authors have studied special numbers and polynomials with their generating functions. Because these special numbers and polynomials including the Bernoulli numbers and polynomials, the Euler numbers and polynomials, the Stirling numbers, the Milne–Thomson numbers and polynomials, the Hermite numbers and polynomials, Central factorial numbers, Cauchy numbers, and the others have many applications not only in mathematics, but also in other related areas. It is well-known that there are also many combinatorial interpretations of these special numbers especially, the Stirling numbers and the central factorial numbers in partition theory, in set theory, in probability theory and in other sciences. For combinatorial interpretations of these special numbers and polynomials with their generating functions see for details [1–31], and the references therein.

In this paper the following notation is used.

$\mathbb{N}=\{1,2,3,\ldots\}$, $\mathbb{N}_{0}=\{0,1,2,3,\ldots\}= \mathbb{N}\cup\{0\}$ and $\mathbb{Z}$ denotes the set of integers, $\mathbb{R}$ denotes the set of real numbers and $\mathbb{C}$ denotes the set of complex numbers. Assuming that $\ln(z)$ denotes the principal branch of the multi-valued function $\ln(z)$ with the imaginary part $\operatorname{Im} ( \ln (z) ) $ constrained by $-\pi<\operatorname{Im} ( \ln(z) ) \leq\pi$. Furthermore, $0^{n}=1$ if $n=0$, and, $0^{n}=0$ if $n\in\mathbb{N}$.

$$ \left ( \begin{matrix} x \\ v\end{matrix} \right ) = \frac{x(x-1)\cdots(x-v+1)}{v!}=\frac{(x)_{v}}{v!} $$

(*cf*. [1–31], and the references therein).

In order to prove identities, relations, formulas, and combinatorial sums related to the special numbers and polynomials of this paper, we need the following generating functions for these special numbers and polynomials including some basic properties of them.

The Bernoulli polynomials are defined by

1 $$ F_{B}(t,x)=\frac{te^{xt}}{e^{t}-1}=\sum_{n=0}^{\infty}B_{n}(x) \frac {t^{n}}{n!}. $$

Substituting $x=0$ into (1), we have the Bernoulli numbers $B_{n}$:

$$ B_{n}=B_{n}(0). $$

The Euler polynomials are defined by

2 $$ F_{E}(t,x)=\frac{2e^{xt}}{e^{t}+1}=\sum_{n=0}^{\infty}E_{n}(x) \frac {t^{n}}{n!}. $$

Substituting $x=0$ into (2), we have the Euler numbers $E_{n}$:

$$ E_{n}=E_{n}(0) $$

(*cf*. [1–31], and the references therein).

The array polynomials are defined by

3 $$ F_{A}(t,x,v)=\frac{ ( e^{t}-1 ) ^{v}e^{xt}}{v!}=\sum_{n=0}^{\infty }S_{v}^{n}(x) \frac{t^{n}}{n!}, $$

where $v\in\mathbb{N}_{0}$. By (3), we have

$$ S_{v}^{n}(x)=\frac{1}{v!}\sum _{j=0}^{v}(-1)^{v-j}\left ( \begin{matrix} v \\ j\end{matrix} \right ) ( x+j ) ^{n} $$

(*cf*. [1, 3, 24, 26]). Substituting $x=0$ into (3), we have the Stirling numbers of the second kind $S_{2}(n,v)$:

$$ S_{2}(n,v):=S_{v}^{n}(0) $$

which defined by means of the following generating function:

4 $$ F_{S}(t,v)=\frac{ ( e^{t}-1 ) ^{v}}{v!}=\sum_{n=0}^{\infty }S_{2}(n,v) \frac{t^{n}}{n!}, $$

where $v\in\mathbb{N}_{0}$ (*cf*. [3, 5, 7, 24, 30, 31] and the references therein).

The Stirling numbers of the first kind are defined by the following generating function:

5 $$ F_{S1}(t,v)=\frac{ ( \log(1+t) ) ^{v}}{v!}=\sum_{n=0}^{\infty }S_{1}(n,v) \frac{t^{n}}{n!}, $$

where $v\in\mathbb{N}_{0}$ (cf. [1–31], and the references therein).

The central factorial numbers $T(n,k)$ of the second kind are defined by means of the following generating function:

6 $$ F_{T}(t,k)=\frac{1}{(2k)!} \bigl( e^{t}+e^{-t}-2 \bigr) ^{k}=\sum_{n=k}^{\infty}T(n,k) \frac{t^{2n}}{ ( 2n ) !}. $$

By using (6), for *n*, $k\in\mathbb{N}$, $T(0,k)=T(n,0)=0$, $T(n,1)=1$ and also

$$ T(n,k)=\frac{1}{(2k)!}\sum_{j=0}^{2k}(-1)^{j} \left ( \begin{matrix} 2k \\ j\end{matrix} \right ) (j-k)^{2n} $$

(*cf*. [2, 4, 5, 26, 32], and the references therein).

Two-variable Hermite polynomials are defined by

7 $$ F(t,x,y:v)=e^{xt+yt^{v}}=\sum_{n=0}^{\infty}H_{n}^{ ( v ) } ( x,y ) \frac{t^{n}}{n!} $$

(*cf*. [7, 20, 22]).

Let *v* be integer $v\geq2$. The polynomials $H_{n}^{ ( v ) } ( x,y ) $ are given by the following explicit formula:

8 $$ H_{n}^{(v)} ( x,y ) =n!\sum_{r=0}^{[\frac{n}{v}]} \frac {x^{n-vr}y^{r}}{r! ( n-vr ) !} $$

(*cf*. [7, 20, 22]). Substituting $y=-1$ and $v=2$ into (8), we have classical Hermite polynomials

$$ H_{n} ( x ) =H_{n}^{(2)} ( x,-1 ) $$

(*cf*. [7, 20, 22]).

The Hermite numbers are defined by

9 $$ F_{H}(t)=e^{-t^{2}}=\sum_{n=0}^{\infty}H_{n} \frac{t^{n}}{n!} $$

(*cf*. [7, 10, 20, 22]).

Three-variable polynomials $y_{6} ( n;x,y,z;a,b,v ) $ are defined by the first author [29] as follows:

10 $$ \mathcal{G} ( t,x,y,z;a,b,v ) = \bigl( b+f ( t,a ) \bigr) ^{z}e^{xt+yh ( t,v ) }= \sum_{n=0}^{\infty}y_{6} ( n;x,y,z;a,b,v ) \frac{t^{n}}{n!}, $$

where $f ( t,a ) $ is a member of family of analytic functions or meromorphic functions, *a* and *b* are any real numbers, *v* is positive integer. When $x=0$, $y=z=1$, Equation (10) reduces to the following numbers:

$$ y_{6} ( n;0,1,1;a,b,v ) =y_{6} ( n;a,b,v ) $$

which defined by means of the following generating function:

$$ \mathcal{J} ( t;a,b,v ) = \bigl( b+f ( t,a ) \bigr) e^{h ( t,v ) }=\sum _{n=0}^{\infty}y_{6} ( n;a,b,v ) \frac{t^{n}}{n!} $$

(*cf*. [29]). When $b=0$, in the numbers $y_{6} ( n;a,b,v ) $ reduce to the Milne–Thomson numbers of order *a*, $\phi _{n}^{(a)}$:

$$ y_{6} ( n;a,0,v ) =\phi_{n}^{(a)} $$

(*cf*. [19, p. 514, Eq-(2)], [9, 29]). Substituting $z=1$ and $b=0$ into (10), we have

$$ y_{6} ( n;x,y,1;a,0,v ) =\Psi_{n}^{(a)}(x,y,v) $$

which is related to a relation between the $y_{6} ( n;x,y,z;a,b,v ) $ and the generalized Milne–Thomson’s polynomials [6]. Setting $z=1$, $b=0$, $y=1$ and $h ( t,0 ) =g ( t ) $ into (10), we also have a relation between the $y_{6} ( n;x,y,z;a,b,v ) $ and the Milne–Thomson polynomials $\Phi _{n}^{(a)}(x)$:

$$ y_{6} ( n;x,1,1;a,0,0 ) =\Phi_{n}^{(a)}(x) $$

(*cf*. [19, 29]). When $z=1$, $b=0$, $y=1$, $f ( t,a ) =1$ and $h ( t,v ) =-\frac{vt^{2}}{2}$, Equation (10) reduces to the Hermite polynomials $H_{n}^{ ( v ) } ( x ) $:

$$ y_{6} ( n;x,1,1;a,0,v ) =H_{n}^{ ( v ) } ( x ) $$

(*cf*. [10, 20, 22, 29]) and also the Hermite numbers

$$ y_{6} ( 2n;0,1,1;a,0,2 ) =H_{2n}=\frac{ ( -1 ) ^{n} ( 2n ) !}{n!} $$

for $n\geq0$ (*cf*. [10, 20, 22]).

### *p*-adic integral

Here, we survey some properties of the *p*-adic integral. Thus, we give some notations and definitions. $\mathbb{Z}_{p}$ denotes the set of *p*-adic integers, $\mathbb{Q}_{p}$ denotes the set of *p*-adic rational numbers, $\mathbb{K}$ denotes a field with a complete valuation and $\mathbb{C}_{p}$ is completion of the algebraic closure of $\mathbb{Q}_{p}$. $C^{1}(\mathbb{Z}_{p}\rightarrow\mathbb{K)}$ denotes the set of continuous derivative functions. Let $f(x)\in C^{1}(\mathbb{Z}_{p}\rightarrow\mathbb{K)}$. Kim [14] defined the *p*-adic *q*-integral as follows:

11 $$ I_{q}\bigl(f(x)\bigr)= \int_{\mathbb{Z}_{p}}f(x)\,d\mu_{q}(x)=\lim_{N\rightarrow \infty} \frac{1}{[p^{N}]_{q}}\sum_{x=0}^{p^{N}-1}f(x)q^{x}, $$

where $q\in\mathbb{C}_{p}$ with $\vert 1-q \vert _{p}<1$, $f\in C^{1}(\mathbb{Z}_{p}\rightarrow\mathbb{K)}$,

$$ [ x ] = [ x:q ] = \textstyle\begin{cases} \frac{1-q^{x}}{1-q},&q\neq1, \\ x,&q=1\end{cases} $$

and

$$ \mu_{q}(x)=\mu_{q} \bigl( x+p^{N} \mathbb{Z}_{p} \bigr) =\frac {q^{x}}{ [ p^{N} ] } $$

is a *q*-distribution on $\mathbb{Z}_{p}$ (*cf*. [14]).

#### Remark 1

If $q\rightarrow1$, then (11) reduces to the Volkenborn integral (or so-called the bosonic integral):

12 $$ \lim_{q\rightarrow1}I_{q}\bigl(f(x)\bigr)=I_{1} \bigl(f(x)\bigr)= \int_{\mathbb{Z}_{p}}f ( x ) \,d\mu_{1} ( x ) =\underset{N \rightarrow \infty}{\lim}\frac{1}{p^{N}}\sum_{x=0}^{p^{N}-1}f ( x ) , $$

where

$$ \mu_{1} ( x ) =\mu_{1} \bigl( x+p^{N} \mathbb{Z}_{p} \bigr) =\frac{1}{p^{N}} $$

denotes the Haar distribution on $\mathbb{Z}_{p}$ (*cf*. [23]), and the references therein).

#### Remark 2

If $q\rightarrow-1$, then (11) reduces to the fermionic *p*-adic integral:

13 $$ \lim_{q\rightarrow-1}I_{q}\bigl(f(x)\bigr)=I_{-1} \bigl(f(x)\bigr)= \int_{\mathbb{Z}_{p}}f ( x ) \,d\mu_{-1} ( x ) =\underset{N \rightarrow \infty}{\lim}\sum_{x=0}^{p^{N}-1} ( -1 ) ^{x}f ( x ) $$

and

$$ \mu_{-1} ( x ) =\mu_{-1} \bigl( x+p^{N} \mathbb{Z}_{p} \bigr) =\frac{(-1)^{x}}{p^{N}} $$

(*cf*. [13]).

The Bernoulli numbers are also given by the following bosonic *p*-adic integral:

14 $$ B_{n}= \int_{\mathbb{Z}_{p}}x^{n}\,d\mu_{1} ( x ) $$

(*cf*. [14, 23]).

On the other hand, the Euler numbers are also given by the following fermionic *p*-adic integral:

15 $$ E_{n}= \int_{\mathbb{Z}_{p}}x^{n}\,d\mu_{-1} ( x ) $$

(*cf*. [13]).

The Daehee numbers $D_{n}$ are introduced by the following bosonic *p*-adic integral:

16 $$ D_{n}= \int_{\mathbb{Z}_{p}} ( x ) _{n}\,d\mu_{1} ( x ) =\sum _{k=0}^{n}S_{1}(n,k)B_{k}= \frac{(-1)^{n}n!}{n+1} $$

(*cf*. [8, 11], [21, p. 45], and the references therein).

The Changhee numbers $\mathit{Ch}_{n}$ are introduced by the following fermionic *p*-adic integral:

17 $$ \mathit{Ch}_{n}= \int_{\mathbb{Z}_{p}} ( x ) _{n}\,d\mu_{-1} ( x ) =\sum _{k=0}^{n}S_{1}(n,k)E_{k}=(-1)^{n}2^{-n}n! $$

(*cf*. [12–14], and the references therein).

We now summarize the results of this paper as follows:

In Sect. 2, by using generating functions and their functional equations, we give some identities including the three-variable polynomials $y_{6} ( n;x,y,z;a,b,v ) $, the Hermite polynomials, the array polynomials and the Stirling numbers of the second kind.

In Sect. 3, by using *p*-adic integrals, we give some identities, combinatorial sums and relations related to the three-variable polynomials $y_{6} ( n;x,y,z;a,b,v ) $, the Bernoulli numbers, the Euler numbers, the Stirling numbers, the Cauchy numbers (or the Bernoulli numbers of the second kind) and other special numbers such as the Daehee numbers and the Changhee numbers.

In Sect. 4, by using generating functions associated with trigonometric functions and the central factorial numbers of the second kind, we derive identities related to the central factorial numbers of the second kind, the array polynomials, and combinatorial sum.

## Identities related to the Hermite polynomials, array polynomials and Stirling numbers of the second kind: generating functions and their functional equations approach

In this section, by applying generating functions and their functional approach, we derive some identities including the three-variable polynomials $y_{6} ( n;x,y,z;a,b,v ) $, the Hermite polynomials, the array polynomials and the Stirling numbers of the second kind.

### Theorem 1

18 $$ y_{6} ( n;x,y,1;a,0,v ) =n!\sum_{j=0}^{ [ \frac {n}{v} ] } \frac{S_{a}^{n-vj}(x)y^{j}}{j!(n-vj)!}, $$

*where* $[ x ] $ *denotes the greatest integer function*.

### Proof

Substituting $b=0$, $h ( t,v ) =t^{v}$and $f ( t,a ) =\frac{1}{a!} ( e^{t}-1 ) ^{a}$ with $a\in\mathbb{N}_{0}$ and $z=1$ into (10), we get the following functional equation:

$$ \mathcal{G} ( t,x,y,1;a,0,v ) =e^{yt^{v}}F_{A}(t,x,v). $$

Combining the above equation with (10), and (3), we obtain

$$ \sum_{n=0}^{\infty}y_{6} ( n;x,y,1;a,0,v ) \frac{t^{n}}{n!}=\sum_{n=0}^{\infty}S_{a}^{n}(x) \frac{t^{n}}{n!}\sum_{n=0}^{\infty } \frac{y^{n} t^{vn}}{n!}. $$

Now, combining the above equation with the following well-known series identity:

$$ \sum_{n=0}^{\infty}\sum _{k=0}^{\infty}A(n,k)=\sum_{n=0}^{\infty } \sum_{k=0}^{ [ \frac{n}{v} ] }A(n,n-vk) $$

(*cf*. [20, Lemma 11, Eq-(7)]), we get

$$ \sum_{n=0}^{\infty}y_{6} ( n;x,y,1;a,0,v ) \frac{t^{n}}{n!}=\sum_{n=0}^{\infty} \Biggl( n!\sum_{j=0}^{ [ \frac{n}{v} ] }\frac{S_{a}^{n-vj}(x)y^{j}}{j!(n-vj)!} \Biggr) \frac{t^{n}}{n!}. $$

Equating the coefficients of $\frac{t^{n}}{n!}$ on both sides of the equation, we arrive at the desired result. □

### Theorem 2

19 $$ y_{6} ( n;x,y,1;a,0,v ) =\sum_{j=0}^{n} \left ( \begin{matrix} n \\ j\end{matrix} \right ) S_{2}(n-j,a)H_{j}^{(v)}(x,y). $$

### Proof

Proof of this theorem was also given in [33, Theorem 2]. We now briefly give another proof of this theorem. Substituting $b=0$, $h ( t,v ) =t^{v}$and $f ( t,a ) =\frac{1}{a!} ( e^{t}-1 ) ^{a}$ with $a\in\mathbb{N}_{0}$ and $z=1$ into (10), we construct the following functional equation:

$$ \mathcal{G} ( t,x,y,1;a,0,v ) =F(t,x,y:v)F_{S}(t,a). $$

Combining the above equation with (10), (7), and (4), we get

$$ \sum_{n=0}^{\infty}y_{6} ( n;x,y,1;a,0,v ) \frac{t^{n}}{n!}=\sum_{n=0}^{\infty}S_{2}(n,a) \frac{t^{n}}{n!}\sum_{n=0}^{\infty }H_{n}^{(v)}(x,y) \frac{t^{n}}{n!}. $$

Therefore

$$ \sum_{n=0}^{\infty}y_{6} ( n;x,y,1;a,0,v ) \frac{t^{n}}{n!}=\sum_{n=0}^{\infty} \left ( \sum_{j=0}^{n}\left ( \begin{matrix} n \\ j\end{matrix} \right ) S_{2}(n-j,a)H_{j}^{(v)}(x,y) \right ) \frac{t^{n}}{n!}. $$

Equating the coefficients $\frac{t^{n}}{n!}$ on both sides of the equation, we arrive at the desired result. □

Combining (18) and (19), we arrive at the following theorem.

### Theorem 3

$$ \sum_{j=0}^{n}\left ( \begin{matrix} n \\ j\end{matrix} \right ) S_{2}(n-j,a)H_{j}^{(v)}(x,y)=n! \sum_{j=0}^{ [ \frac {n}{v} ] }\frac{S_{a}^{n-vj}(x)y^{j}}{j!(n-vj)!}. $$

## Identities and relations related to Stirling numbers and other special numbers: *p*-adic integral approach

In this section, by applying *p*-adic integrals approach, we derive some identities, combinatorial sums and relations related to the three-variable polynomials $y_{6} ( n;x,y,z;a,b,v ) $, the Bernoulli numbers, the Euler numbers, the Stirling numbers, the Cauchy numbers (or the Bernoulli numbers of the second kind) and other special numbers such as the Daehee numbers and the Changhee numbers.

### Theorem 4

*Let* $m\in\mathbb{N}_{0}$ *and* $z\in\mathbb{R}$. *We have*

20 $$ y_{6} ( m;0,0,z;1,1,1 ) =\sum_{n=0}^{m}S_{2}(m,n) (z)_{n}=z^{m}. $$

### Proof

Substituting $a=b=v=1$, $x=y=0$ and $f ( t,1 ) =e^{t}-1$ into (10), we get

$$ \mathcal{G} ( t,0,0,z;1,1,1 ) = \bigl( 1+\bigl(e^{t}-1\bigr) \bigr) ^{z}=\sum_{n=0}^{\infty}y_{6} ( n;0,0,z;1,1,1 ) \frac{t^{n}}{n!}. $$

We assume that $\vert e^{t}-1 \vert <1$. We obtain

$$ \sum_{m=0}^{\infty}y_{6} ( m;0,0,z;1,1,1 ) \frac{t^{m}}{m!}=\sum_{n=0}^{\infty}(z)_{n} \frac{(e^{t}-1)^{n}}{n!}, $$

where

$$ (z)_{n}=z(z-1)\cdots(z-n+1). $$

Combining the above equation with (4), since $S_{2}(m,n)=0$ for $m< n $, we obtain

$$ \sum_{m=0}^{\infty}y_{6} ( m;0,0,z;1,1,1 ) \frac{t^{m}}{m!}=\sum_{m=0}^{\infty} \Biggl( \sum_{n=0}^{m}S_{2}(m,n) (z)_{n} \Biggr) \frac{t^{m}}{m!}. $$

Equating the coefficients of $\frac{t^{m}}{m!}$ on both sides of the equation, we arrive at the desired result. □

It is time to give integral formulas for the three-variable polynomials $y_{6} ( n;x,y,z;a,b,v ) $.

By applying the bosonic *p*-adic integral (or the Volkenborn integral) to (20), we obtain

$$ \int_{\mathbb{Z}_{p}}y_{6} ( m;0,0,z;1,1,1 ) \,d\mu _{1} ( z ) =\sum_{n=0}^{m}S_{2}(m,n) \int_{\mathbb{Z}_{p}} ( z ) _{n}\,d\mu_{1} ( z ). $$

By combining the above equation with (16), we obtain the following *p*-adic integral formulas, respectively:

21 $$ \begin{gathered} \int_{\mathbb{Z}_{p}}y_{6} ( m;0,0,z;1,1,1 ) \,d\mu _{1} ( z ) =\sum_{n=0}^{m}S_{2}(m,n)D_{n}, \\ \int_{\mathbb{Z}_{p}}y_{6} ( m;0,0,z;1,1,1 ) \,d\mu _{1} ( z ) =\sum_{n=0}^{m} ( -1 ) ^{n}\frac {n!S_{2}(m,n)}{n+1}, \end{gathered} $$

and we also have

22 $$ \int_{\mathbb{Z}_{p}}y_{6} ( m;0,0,z;1,1,1 ) \,d\mu _{1} ( z ) =\sum_{n=0}^{m}\sum _{j=0}^{n}S_{2}(m,n)S_{1}(n,j)B_{j}. $$

Combining Eqs. (21) and (22), we get the following theorem.

### Theorem 5

*Let* $m\in\mathbb{N}_{0}$. *We have*

23 $$ \sum_{n=0}^{m}\sum _{j=0}^{n}S_{2}(m,n)S_{1}(n,j)B_{j}= \sum_{n=0}^{m} ( -1 ) ^{n} \frac{n!S_{2}(m,n)}{n+1}. $$

We observe that, using the orthogonality relation of the Stirling numbers, Eq. (23) reduces to the following well-known relations for the Bernoulli numbers:

$$ B_{m}=\sum_{n=0}^{m} ( -1 ) ^{n}\frac{n!S_{2}(m,n)}{n+1} $$

(*cf*. [3, 7, 11, 17, 18, 25], and the references therein).

By applying the fermionic *p*-adic integral to (20), we obtain

$$ \int_{\mathbb{Z}_{p}}y_{6} ( m;0,0,z;1,1,1 ) \,d\mu _{-1} ( z ) =\sum_{n=0}^{m}S_{2}(m,n) \int_{\mathbb{Z}_{p}} ( z ) _{n}\,d\mu_{-1} ( z ). $$

By combining the above equation with (17), we obtain

24 $$ \int_{\mathbb{Z}_{p}}y_{6} ( m;0,0,z;1,1,1 ) \,d\mu _{-1} ( z ) =\sum_{n=0}^{m} ( -1 ) ^{n}\frac {n!}{2^{n}}S_{2}(m,n), $$

and we also obtain

25 $$ \int_{\mathbb{Z}_{p}}y_{6} ( m;0,0,z;1,1,1 ) \,d\mu _{-1} ( z ) =\sum_{n=0}^{m}\sum _{j=0}^{n}S_{2}(m,n)S_{1}(n,j)E_{j}. $$

Combining the above equations, we get the following theorem.

### Theorem 6

*Let* $m\in\mathbb{N}_{0}$. *We have*

26 $$ \sum_{n=0}^{m}\sum _{j=0}^{n}S_{2}(m,n)S_{1}(n,j)E_{j}= \sum_{n=0}^{m} ( -1 ) ^{n} \frac{n!}{2^{n}}S_{2}(m,n). $$

We also observe that, using the orthogonality relation of the Stirling numbers, Eq. (26) reduces to the following well-known relations for the Bernoulli numbers:

$$ E_{m}=\sum_{n=0}^{m} ( -1 ) ^{n}\frac{n!}{2^{n}}S_{2}(m,n) $$

(*cf*. [7, 12, 17, 18, 25], and the references therein).

Integrating Eq. (20) with respect to *z* from 0 to 1, we obtain

$$ \int_{0}^{1}y_{6} ( m;0,0,z;1,1,1 ) \,dz= \sum_{n=0}^{m}S_{2}(m,n) \int_{0}^{1} ( z ) _{n}\,dz. $$

Combining the above integral equation with the integral equation for the Cauchy numbers (the Bernoulli numbers of the second kind),

$$ b_{n}(0)= \int_{0}^{1} ( z ) _{n}\,dz $$

(*cf*. [22, p. 114]), we get the following integral representation of the polynomials $y_{6} ( m;0,0,z;1,1,1 ) $.

### Corollary 1

*Let* $m\in\mathbb{N}_{0}$. *We have*

$$ \int_{0}^{1}y_{6} ( m;0,0,z;1,1,1 ) \,dz= \sum_{n=0}^{m}S_{2}(m,n)b_{n}(0). $$

## Identities including the central factorial numbers of the second kind and array polynomials

In this section, by using special infinite series including trigonometric functions and the central factorial numbers of the second kind, we derive identities associated with the central factorial numbers of the second kind and the array polynomials.

In [2, Theorem 4.1.1 and Proposition 4.1.5], Butzer et al. gave the following alternative generating functions for the central factorial numbers of the second kind:

27 $$\begin{aligned}& \frac{1}{(2m)!} \biggl( 2\sin \biggl( \frac{t}{2} \biggr) \biggr) ^{2m}=\sum_{n=0}^{\infty}(-1)^{n+m}T(2n,2m) \frac{t^{2n}}{(2n)!} , \end{aligned}$$

28 $$\begin{aligned}& \biggl( \cos \biggl( \frac{t}{2} \biggr) \biggr) ^{2m}=\sum _{n=0}^{\infty}(-1)^{n+m}T(2n,2m) \frac{t^{2n}}{(2n)!}\sum_{j=0}^{m}\left ( \begin{matrix} m \\ j\end{matrix} \right ) 4^{-j}(2j)!T(2n,2j), \end{aligned}$$

$m\in\mathbb{N}_{0}$.

Since

$$ \sin ( t ) =\frac{e^{it}-e^{-it}}{2i}, $$

where $i^{2}=-1$, after some elementary calculations, we obtain

29 $$ \bigl( \sin ( t ) \bigr) ^{2m}=(2m)!\sum_{n=0}^{\infty }(-1)^{n-m}(i)^{n}2^{n-2m}S_{2m}^{n}(-m) \frac{t^{n}}{n!}. $$

Combining (27) and (28), and also using the Cauchy product formula for a series product, we also obtain

30 $$ \begin{aligned}[b] \biggl( 2\sin \biggl( \frac{t}{2} \biggr) \cos \biggl( \frac{t}{2} \biggr) \biggr) ^{2m}&=\sum_{n=0}^{\infty} \sum_{v=0}^{n}(-1)^{n+m}\left ( \begin{matrix} 2n \\ 2v\end{matrix} \right ) (2m)! \\ &\quad {} \times T(2n-2v,2m) \sum_{j=0}^{m} \left ( \begin{matrix} m \\ j\end{matrix} \right ) 4^{-j}(2j)!T(2v,2j)\frac{t^{2n}}{(2n)!}. \end{aligned} $$

Since

$$ 2\sin \biggl( \frac{t}{2} \biggr) \cos \biggl( \frac{t}{2} \biggr) =\sin ( t ) , $$

combining (29) and (30), after some calculations, we arrive at the following theorem including a relation between the central factorial numbers of the second kind and the array polynomials.

### Theorem 7

*For* $m,n\in\mathbb{N}_{0}$ *we have*

$$ S_{2m}^{2n}(-m)=\sum_{v=0}^{n} \left ( \begin{matrix} 2n \\ 2v\end{matrix} \right ) T(2n-2v,2m)\sum_{j=0}^{m}\left ( \begin{matrix} m \\ j\end{matrix} \right ) 4^{n-m-j}(2j)!T(2v,2j). $$

By combining (29) and (30), we also get following corollary.

### Corollary 2

*For* $m,n\in\mathbb{N}_{0}$ *we have*

31 $$ S_{2m}^{2n+1}(-m)=0. $$

With the help of Eq. (31), we also obtain the following combinatorial sum.

### Corollary 3

*For* $m,n\in\mathbb{N}_{0}$ *we have*

$$ \sum_{j=0}^{2m}\sum _{k=0}^{2n+1}(-1)^{-j-k}\left ( \begin{matrix} 2m \\ j\end{matrix} \right ) \left ( \begin{matrix} 2n+1 \\ k\end{matrix} \right ) j^{k}m^{2n+1-k}=0. $$

## Conclusion

This paper contains many kind of identities and relations related to the Milne–Thomson polynomials, the Hermite polynomials, the Bernoulli numbers, the Euler numbers, the Stirling numbers, the central factorial numbers, and the Cauchy numbers. By applying not only *p*-adic integral, but also the Riemann integral methods, many identities relations and formulas related to the aforementioned numbers and polynomials, and also the combinatorial sums are given. By using the orthogonality relation of the Stirling numbers, explicit formulas for the Bernoulli numbers and the Euler numbers are provided.

The results of this paper have potential applicability to physics, engineering and other related fields, especially branches of mathematics.
